# Convergent evolution of plant specialized 1,4-naphthoquinones: metabolism, trafficking, and resistance to their allelopathic effects

**DOI:** 10.1093/jxb/eraa462

**Published:** 2020-12-01

**Authors:** George W Meyer, Maria A Bahamon Naranjo, Joshua R Widhalm

**Affiliations:** 1 Department of Horticulture and Landscape Architecture, Purdue University, IN, USA; 2 Purdue Center for Plant Biology, Purdue University, West Lafayette, IN, USA; 3 The James Hutton Institute, UK

**Keywords:** Allelopathy, convergent evolution, juglone, 1,4-naphthoquinone, shikonin, specialized metabolism

## Abstract

Plant 1,4-naphthoquinones encompass a class of specialized metabolites known to mediate numerous plant–biotic interactions. This class of compounds also presents a remarkable case of convergent evolution. The 1,4-naphthoquinones are synthesized by species belonging to nearly 20 disparate orders spread throughout vascular plants, and their production occurs via one of four known biochemically distinct pathways. Recent developments from large-scale biology and genetic studies corroborate the existence of multiple pathways to synthesize plant 1,4-naphthoquinones and indicate that extraordinary events of metabolic innovation and links to respiratory and photosynthetic quinone metabolism probably contributed to their independent evolution. Moreover, because many 1,4-naphthoquinones are excreted into the rhizosphere and they are highly reactive in biological systems, plants that synthesize these compounds also needed to independently evolve strategies to deploy them and to resist their effects. In this review, we highlight new progress made in understanding specialized 1,4-naphthoquinone biosynthesis and trafficking with a focus on how these discoveries have shed light on the convergent evolution and diversification of this class of compounds in plants. We also discuss how emerging themes in metabolism-based herbicide resistance may provide clues to mechanisms plants employ to tolerate allelopathic 1,4-naphthoquinones.

## Teaser

We highlight how recent discoveries have revealed insight into the evolution of plant specialized 1,4-naphthoquinone metabolism and trafficking, and offer a perspective for investigating mechanisms plants use to tolerate allelopathic 1,4-naphthoquinones.

## Introduction

Plants produce an amazing diversity of specialized metabolites playing roles in adapting to ecological niches. Many of the studied compounds fall within the ‘major’ chemical classes of specialized metabolites: the phenylpropanoids/benzenoids (Vogt, 2010; Widhalm and Dudareva, 2015), terpenoids (Nagegowda and Gupta, 2020), and alkaloids (Ziegler and Facchini, 2008). With the explosion of functional and comparative genomics and advances in metabolomics, however, there has been major progress in understanding the evolution of lesser studied chemical classes across plant taxa (Smith *et al.*, 2019). One such class is the specialized 1,4-naphthoquinones, which has gained interest due to the potential of its members to serve as novel therapeutics (Duru *et al.*, 2014), drug scaffolds (F. Wang *et al.*, 2019), and leads for natural product-based herbicides (Durán *et al.*, 2018).

**Fig. 1.**
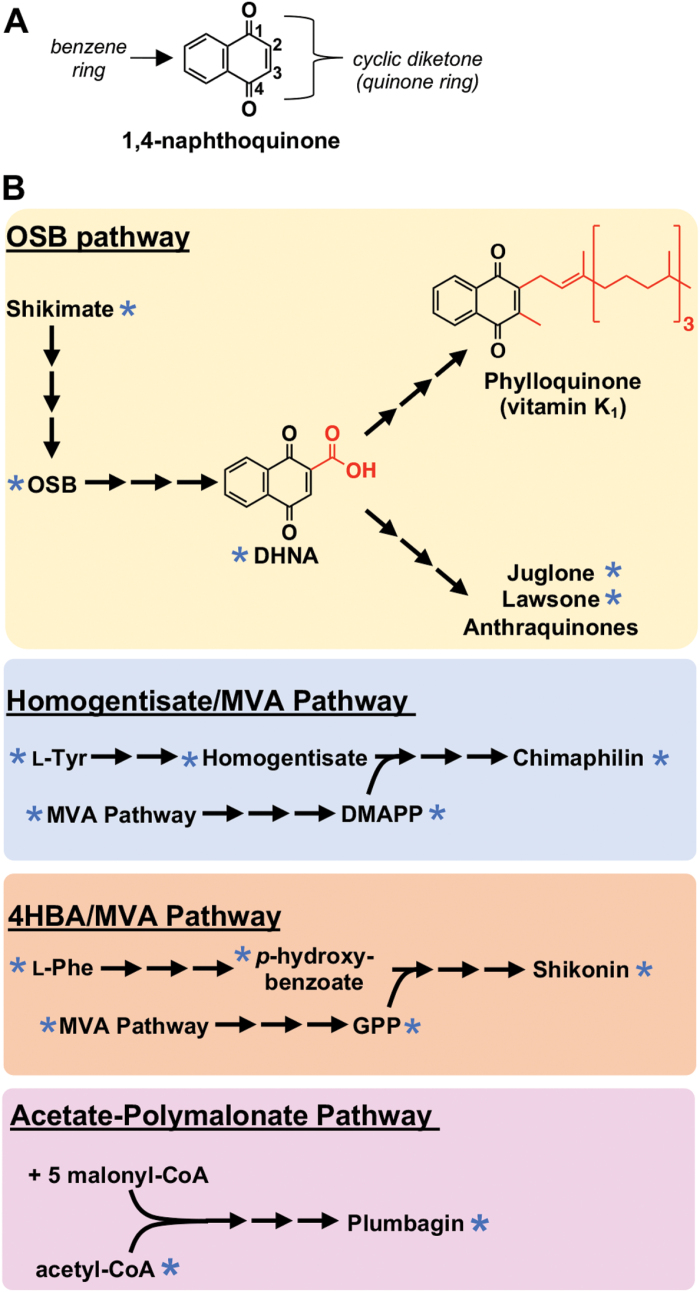
Core structure (A) and known biosynthetic pathways (B) of plant 1,4-naphthoquinones. Asterisks indicate support from labeling studies as detailed in the review by Widhalm and Rhodes (2016). Abbreviations: DHNA, 1,4-dihyroxynaphthoic acid; DMAPP, dimethylallyl diphosphate; OSB, *o*-succinylbenzoic acid.

The 1,4-naphthoquinones are a diverse group of compounds structurally related to naphthalene. The core 1,4-naphthoquinone skeleton is comprised of a benzene ring fused to a fully conjugated cyclic diketone with *para*-oriented carbonyl groups (Fig. 1A). All plants synthesize phylloquinone (vitamin K_1_), a methylated and phytylated 1,4-naphthoquinone (Fig. 1B) that functions as a one-electron transporter at the A1 site of PSI and as a two-electron acceptor from a protein disulfide isomerase involved in folding plastidial proteins (van Oostende *et al.*, 2011). While phylloquinone is anchored to thylakoid and plastoglobule membranes by a liposoluble side chain, the hundreds of known specialized 1,4-naphthoquinones collectively produced by plants are untethered, often deployed in the environment, and mediate various plant–microbial, plant–fungal, and plant–insect interactions, as well as plant–plant competition (allelopathy) (Widhalm and Rhodes, 2016). New evidence also implicates them in responding to abiotic stresses, such as iron deficiency (Rajniak *et al.*, 2018).

The bioactivities of 1,4-naphthoquinones are derived from their chemically reactive nature *in vivo*. Unlike higher redox potential quinones, 1,4-naphthoquinones in partial or fully reduced forms spontaneously oxidize in the presence of oxygen. The reactive oxygen species (ROS) generated from this autooxidation oxidatively modify lipids and proteins, causing immediate damage to cells, or lead to production of more damaging free radicals that cause DNA mutations. Depending on the functional groups attached to the naphthoquinone ring, the oxidized quinone itself can form adducts with reduced glutathione (GSH), proteins, and DNA, leading to thiol depletion or macromolecule damage (Klotz *et al.*, 2014). Most prokaryotes today still solely or conditionally rely on 1,4-naphthoquinones (menaquinones; vitamin K_2_) in their anaerobic respiratory electron transport chains (Schoepp-Cothenet *et al.*, 2013). The propensity of 1,4-naphthoquinones to react with oxygen, however, probably drove evolution from ancestral menaquinones to higher redox potential quinones in the bioenergetic chains of some prokaryote lineages following the ‘Great Oxidation Event’ 2.5 billion years ago (Hohmann-Marriott and Blankenship, 2011), including in the progenitors of modern-day mitochondria and chloroplasts (Schoepp-Cothenet *et al.*, 2013). As a result, the present macroscopic biosphere is dominated by Eukaryotes relying on ubiquinone for aerobic respiration and/or plastoquinone for oxygenic photosynthesis to generate ATP (Bergdoll *et al.*, 2016). Despite the shift to high redox potential quinones, many species throughout the tree of life, and in the plant kingdom in particular, appear to have convergently evolved to produce specialized 1,4-naphthoquinones that function as novel ‘chemical weapons’ in an oxygenic environment.

Convergent evolution in plant metabolism is surprisingly common (Pichersky and Lewinsohn, 2011). There are numerous examples of plants—mostly Angiosperms—belonging to different lineages that independently evolved to produce the same compound (e.g. caffeine; Huang *et al.*, 2016) or structurally similar compounds (e.g. stilbenes; Tropf *et al.*, 1994), or of plants in disparate lineages that synthesize different compounds to fulfill the same function (e.g. floral volatiles; Dudareva *et al.*, 2013). With recent progress in understanding plant 1,4-naphthoquinone distribution, metabolism, and function, it is becoming evident that the ability to synthesize these compounds has independently evolved several times and was facilitated by extraordinary events of metabolic innovation and/or metabolic links to respiratory and photosynthetic quinone metabolism (McCoy *et al.*, 2018; Auber *et al.*, 2020; Ueoka *et al.*, 2020). In this review, we highlight advances in understanding specialized 1,4-naphthoquinone biosynthesis, trafficking, and resistance strategies with emphasis on how these discoveries have shed light on the convergent evolution and diversification of this class of compounds in plants.

## Convergent evolution of plant 1,4-naphthoquinone biosynthesis

### Specialized 1,4-naphthoquinones are distributed across multiple discrete plant taxa

**Fig. 2.**
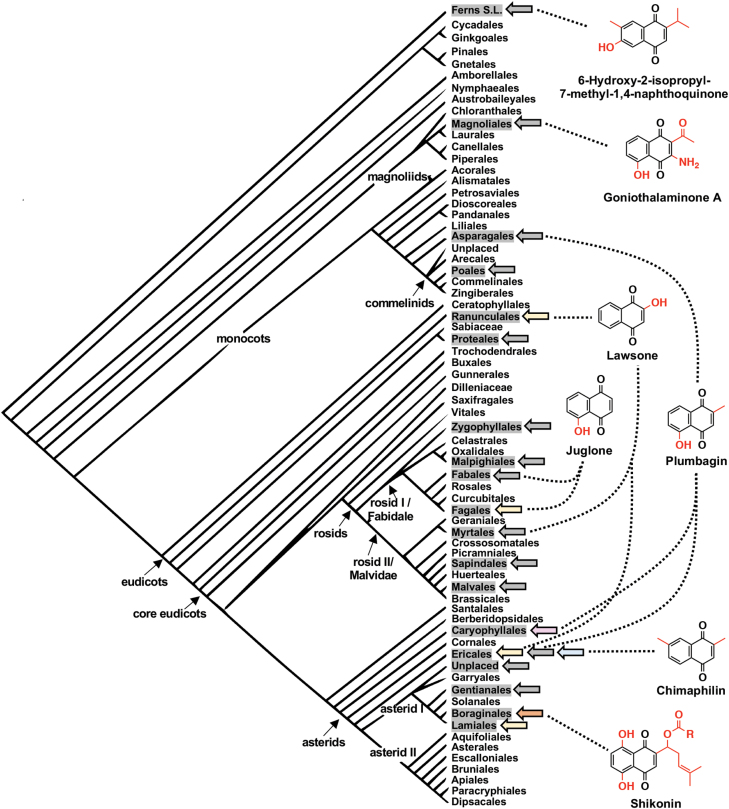
Specialized 1,4-naphthoquinone metabolism is widely distributed across vascular plants. Orders containing species in which specialized 1,4-naphthoquinones have been detected and reported (Hook *et al.*, 2014) are shaded in gray. Examples of 1,4-naphthoquinones observed in ferns, magnoliids, monocots, and dicots are shown. Shaded arrows next to orders indicate experimentally supported metabolic origins (Widhalm and Rhodes, 2016; Rajniak *et al.*, 2018; Auber *et al.*, 2020). Arrow colors depicting metabolic routes are the same as those in Fig. 1B: yellow, OSB pathway; blue, homogentisate/MVA pathway; orange, 4HBA/MVA pathway; magenta, acetate–polymalonate pathway; gray, no experimental evidence for metabolic origin of detected 1,4-naphtoquinones. The phylogenetic reconstruction of plant evolution presented is from the Angiosperm Phylogeny Group (P.F. Stevens, Angiosperm Phylogeny Website, version 14, July 2017; http://www.mobot.org/MOBOT/research/APweb/).

Plotting the distribution of detected 1,4-naphthoquinones (excluding phylloquinone) in plants on the phylogenetic reconstruction of plant evolution provided by the Angiosperm Phylogeny Group shows that the ability to synthesize these compounds is scattered across multiple lineages of vascular plants (Fig. 2). While they appear to be predominantly produced by dicots, specialized 1,4-naphthoquinones are reported in monocots, magnoliids, and ferns. *Lygodium japonicum*, also known as ‘Japanese climbing fern’, is a true fern native to Asia that was introduced into other parts of the world as an ornamental, later escaped, and is today regarded as a pest in many non-native habitats (‘Lygodium japonicum (Thunb.) Sw’, 2019). Investigation into the chemical constituents of *Lygodium* roots, which are used in traditional Chinese medicine, revealed the presence of a new compound, 6-hydroxy-2-isopropyl-7-methyl-1,4-naphthoquinone (Chen *et al.*, 2010) (Fig. 2). Similarly, two new 1,4-naphthoquinones, goniothalaminone A and B, were isolated from the roots of *Goniothalamus scortechinii* (Prawat *et al.*, 2012), a medicinal plant native to Thailand that is a member of the Magnoliales order (Fig. 2). The monocotyledonous species, *Aristea ecklonii* (Asparagales), or ‘blue-eyed iris’, is native to parts of central and southern Africa and produces plumbagin (Fig. 2) in its rhizomes (Kumar *et al.*, 1985). Plumbagin is also produced by dicots such as *Diospyros* species in the Ebenaceae family (Ericales) as well as in members of several families in the Caryophyllales, including carnivorous plant families (Drosophyllaceae, Nepenthaceae, and Droseraceae) and non-carnivorous families such as the Plumbaginaceae (Hook *et al.*, 2014). Plumbagin is not the only structurally identical 1,4-naphthoquinone detected in species belonging to distantly separated orders. Lawsone (Fig. 2), the compound responsible for the orange dyeing properties of henna (*Lawsonia inermis*, Myrtales), was also recently reported in root exudates of opium poppy (*Papaver somniferum*, Ranunculales) (Rajniak *et al.*, 2018). Thus, plumbagin and lawsone are obvious examples of convergently evolved 1,4-naphthoquinones. The wide dispersal of structurally diverse 1,4-naphthoquinones (Fig. 2) and their apparent absence in intervening taxa further suggests that 1,4-naphthoquinone metabolism independently evolved multiple times in plants. However, when determining convergent evolution in plant metabolism, it is important to remember that ‘absence of evidence is not evidence of absence’ (Pichersky and Lewinsohn, 2011). It is possible that 1,4-naphthoquinones are more ubiquitously present in plants than currently understood but at levels below detection or in unexamined tissues, developmental stages, or environmental conditions.

### Plant 1,4-naphthoquinones are biosynthesized via several different pathways

Support for multiple events of convergent evolution becomes stronger when considering that several pathways starting from different metabolic precursors and involving different intermediates and enzymes have been implicated in plant 1,4-naphthoquinone biosynthesis. A review by Widhalm and Rhodes (2016) previously detailed numerous biochemical and tracer studies showing that specialized 1,4-naphthoquinones are synthesized via one of at least four different metabolic routes (Fig. 1B). Since then, several studies have further corroborated the existence of different 1,4-naphthoquinone pathways across plants and provided insight into their metabolic evolution (McCoy *et al.*, 2018; Rajniak *et al.*, 2018; S. Wang *et al.*, 2019; Auber *et al.*, 2020; Song *et al.*, 2020; Ueoka *et al.*, 2020) (Box 1). Moreover, genome assemblies from 1,4-naphthoquinone-producing species also revealed potential glimpses into the metabolic innovation that facilitated 1,4-naphthoquinone pathway evolution. *De novo* assembly of the red gromwell (*Lithospermum erythrorhizon*) and *Echium plantagineum* genomes indicated that retrotransposition-derived duplication (Auber *et al.*, 2020) and whole-genome duplication (Tang *et al.*, 2020) within the Boraginales contributed to establishing the shikonin/alkannin pathway. Reported genome assemblies from English walnut (*Juglans regia*) (Martínez-García *et al.*, 2016), *J. regia*×*J. microcarpa* hybrid (Zhu *et al.*, 2019), and several other *Juglans* species (Stevens *et al.*, 2018) underscored the importance of whole-genome duplication (Luo *et al.*, 2015) in driving metabolic diversification in the Juglandaceae. These genome assemblies are also expected to serve as new resources for gene discovery in 1,4-naphthoquinone metabolism. In the next section, we explore newly uncovered links between 1,4-naphthoquinones and the metabolism of photosynthetic and respiratory quinones that perhaps contributed to the convergent evolution of specialized 1,4-naphthoquinone biosynthesis.

Box 1. **Key developments in understanding metabolic innovation in the evolution of specialized plant 1,4-naphthoquinone metabolism**

- **Retrotransposition-derived duplication contributed to evolution of the shikonin pathway**

*De novo* assembly of the red gromwell (*Lithospermum erythrorhizon*) genome and phylogenetic analysis by Auber *et al.* (2020) showed that *PGT* genes arose in a common ancestor of modern shikonin/alkannin-producing Boraginaceae species via a retrotransposition-derived duplication event and subsequent neofunctionalization of an ancestral prenyltransferase gene.

- **The shikonin pathway relies on GPP derived from the MVA pathway via a unique cytosolic GPPS**

Ueoka *et al.* (2020) showed that a histidine residue adjacent to the first aspartate-rich motif in a novel cytoplasmic farnesyl diphosphate synthase (FPPS) is responsible for the evolved geranyl diphosphate synthase (GPPS) activity in the enzyme recruited to provide geranyl diphosphate (GPP) precursor to the shikonin pathway.

- **Members of the expanded CYP76B subfamily in the Boraginaceae function in the shikonin pathway**

S. Wang *et al.* (2019) showed that CYP76B74, which localizes to the endoplasmic reticulum membrane, catalyzes the hydroxylation of the shikonin pathway intermediate geranylhydroquinone in *Arnebia euchroma*. Song *et al.* (2020) more recently demonstrated that *A. euchroma* CYP76B100 is also capable of hydroxylating geranylhydroquinone, while CYP76B101 catalyzes the three-step oxidation of geranylhydroquinone to form a 3''-carboxylic acid derivative of geranylhydroquinone. The expansion of the CYP76B subfamily in the Boraginaceae thus appears connected to evolution of shikonin biosynthesis.

- **Repeated evolution of 1,4-naphthoquinone pathways from an intermediate of the phylloquinone pathway**

McCoy *et al.* (2018) demonstrated that biosynthesis of juglone in black walnut (*Juglans nigra*) relies on the phylloquinone pathway intermediate 1,4-dihydroxynaphthoic acid (DHNA; Fig. 1B). While using untargeted metabolomics to investigate compounds produced by roots in response to iron deficiency, Rajniak *et al.* (2018) detected lawsone and carboxylated lawsone (3-hydroxy-DHNA) in the root exudates from opium poppy (*Papaver somniferum*). This suggests that like juglone in black walnut (Fagales, Fig. 2), lawsone in opium poppy (Ranunculales, Fig. 2) is synthesized from DHNA. Lawsone is also the 1,4-naphthoquinone responsible for the orange dyeing properties of henna (*Lawsonia inermis*, Myrtales) and was shown through classic tracer studies in *Impatiens* species (Ericales) to be synthesized via DHNA (see text for further details).

### Metabolic connections to primary quinone metabolism

One way in which new specialized metabolic pathways evolve is when a newly acquired enzyme converts an intermediate from an existing pathway into a novel intermediate or end product (Pichersky and Gang, 2000). If that intermediate is ubiquitously present in plants and requires minimal modification to produce a biochemically active compound, it may lead to repeated evolution of functionally and/or structurally similar compounds. Recent studies point to the repeated evolution of specialized 1,4-naphthoquinones from 1,4-dihydroxynaphthoic acid (DHNA), an intermediate in the biosynthesis of phylloquinone (Fig. 1B). McCoy *et al.* (2018) demonstrated that the naphthalenoid moiety of the allelochemical juglone in black walnut (*J. nigra*) originates from DHNA made via the phylloquinone pathway. The recent report of carboxylated lawsone (3-hydroxy-DHNA) in opium poppy root exudates suggests that DHNA is also the precursor for lawsone in this species (Rajniak *et al.*, 2018). These results from black walnut (Fagales) and opium poppy (Ranunculales) validate labeling studies in *Impatiens balsamina* (Zenk and Leistner, 1967; Chung *et al.*, 1994), suggesting that DHNA is the immediate precursor to lawsone in *Impatiens* species (Ericales; Fig. 2). Transcriptomic analysis coupled with untargeted metabolomics in the medicinal plant *Ophiorrhiza pumila* (Gentianales) suggests that DHNA is the precursor for its anthraquinones (Yamazaki *et al.*, 2013). Thus, it seems that DHNA has been recruited on multiple occasions in disparate lineages to provide the precursor for synthesis of plant specialized quinones.

New research also points to a metabolic connection between shikonin and the vital respiratory cofactor ubiquinone. The shikonin and ubiquinone pathways both start with conjugation of *p*-hydroxybenzoate and a polyprenyl diphosphate. Like the ubiquinone pathway (Ducluzeau *et al.*, 2012; Liu and Lu, 2016), the prenyl diphosphate precursor for shikonin biosynthesis, geranyl diphosphate (GPP), is synthesized using the five-carbon building blocks isopentenyl diphosphate (IPP) and dimethylallyl diphosphate (DMAPP) derived from the mevalonic acid (MVA) pathway (Schmid and Zenk, 1971; Gaisser and Heide, 1996). The origin of MVA-derived GPP for shikonin, however, has remained enigmatic as GPP is usually produced in plants from the condensation of IPP and DMAPP derived from the methylerythritol phosphate (MEP) pathway via plastid-localized GPP synthases (GPPSs) (Vranová *et al.*, 2013). In a breakthrough study by Ueoka *et al.* (2020), it was shown that GPP used to synthesize shikonin can be produced by a novel cytoplasmic farnesyl diphosphate synthase (FPPS) that independently evolved GPPS activity. This explains how IPP and DMAPP from the MVA pathway are used to produce GPP, and further validates that shikonin and ubiquinone are derived from the same isoprenoid precursor pool. Moreover, ubiquinone polyprenyltransferases (PPTs) and shikonin *p*-hydroxybenzoate:geranyltransferases (PGTs), which catalyze the conjugation of *p*-hydroxybenzoate with their prenyl diphosphates, were found to belong to the same orthogroup (Auber *et al.*, 2020). Subsequent phylogenetic analysis of prenyltransferases encoded in the *L. erythrorhizon* genome and in other shikonin- and non-shikonin-producing species, indicated that *PGT* genes arose in a common ancestor of modern shikonin/alkannin-producing Boraginaceae species via a retrotransposition-derived duplication event and subsequent neofunctionalization of an ancestral prenyltransferase gene (Auber *et al.*, 2020). Whether the duplication was from a PPT or another related prenyltransferase is unclear. Given that there are other modifications to the *p*-hydroxybenzoate moiety that occur in both ubiquinone and shikonin biosynthesis and *a priori* require the action of similar enzymes, it is possible that there are additional evolutionary linkages between the two pathways (Auber *et al.*, 2020). Like ubiquinone and shikonin, similar connections based on shared precursor pools and metabolic modifications link the synthesis of plastoquinone and the specialized 1,4-naphthoquinone chimaphilin (Fig. 1B) (Widhalm and Rhodes, 2016).

## Plants deploy specialized 1,4-naphthoquinones in different ways

It is increasingly apparent that plants independently evolved diverse mechanisms to release 1,4-naphthoquinones into the rhizosphere. Looking at other plant metabolites, biosynthesis of volatiles, for example, typically occurs in epidermal cells and allows for direct emission into the environment (Widhalm *et al.*, 2015). The same biological principle seems to apply in some cases for excreting 1,4-naphthoquinones. Expression of shikonin metabolic genes is higher and shikonin pool sizes are more abundant in *L. erythrorhizon* root periderm compared with vascular tissue (Auber *et al.*, 2020). This permits direct access for release into the soil. Shikonins have also been found to be more abundant in the periderm of *Echium* species (Durán *et al.*, 2017), as has juglone in the roots of black walnut trees (McCoy *et al.*, 2018). At the subcellular level, multiple mechanisms are implicated in the trafficking of naphthoquinones to the apoplast. Confocal imaging and application of membrane trafficking inhibitors to *L. erythrorhizon* hairy roots suggests that shikonin can be secreted to the apoplast inside endoplasmic reticulum-derived vesicles via trafficking pathways similar to those involved in protein secretion (Tatsumi *et al.*, 2016). At the same time, genetic evidence indicates that excretion of shikonins from *L. erythrorhizon* hairy roots also involves plasma membrane-localized ATP-binding cassette (ABC) transporters (Zhu *et al*., 2017*a*, *b*). In black walnut, it is proposed that arbuscular mycorrhizal fungal hyphae contribute to transporting juglone from roots into the rhizosphere (Achatz and Rillig, 2014; Achatz *et al.*, 2014). Interestingly, new research suggests that some plants have evolved to use aerial organs to deploy 1,4-naphthoquinones into the soil. Block *et al.* (2019) found that lawsone and 2-methoxy-1,4-naphthoquinone are present in the nectar excreted from *Impatiens glandulifera* extrafloral nectaries, which could be the conduit by which the allelochemicals are leached into the soil by rain from above-ground tissues (Ruckli *et al.*, 2014). In *Plagiobothrys arizonicus*, alkannin is visible in the margins and midvein of the abaxial leaf surface and is readily exuded when the leaves are crushed (Kelley, 2012). Hook and Sheridan (2020) showed that cultured plantlets of *Streptocarpus dunnii* excrete a mixture of allelopathic naphtho- and anthraquinones from their fibrous roots as well as from around their petiolodes and leaf bases, which bear glandular trichomes involved in synthesizing (±)-dunnione (Hook *et al.*, 2018). Taken together, it appears that in addition to convergent evolution of 1,4-naphthoquinone biosynthesis, plants have also independently evolved different ways to deploy the compounds into the rhizosphere.

## Metabolism-based resistance to allelopathic 1,4-naphthoquinones in plants

Just as plants independently evolved biosynthesis pathways for 1,4-naphthoquinones and strategies to deploy them, species producing allelopathic 1,4-naphthoquinones must have evolved mechanisms to resist their herbicidal effects. How this is accomplished is poorly understood for specialized 1,4-naphthoquinone metabolism, and allelopathy in general. One potential place to look for clues is in the numerous weed species that evolved resistances to synthetic herbicides (Heap, 2020). Seventy years of synthetic herbicide use has created strong selection pressure to drive evolution of target site resistance (TSR) and non-target site resistance (NTSR). A recent review by Gaines *et al.* (2020) provides an excellent update on the contributions of TSR, NTSR, and the combination of both to the evolutionary resilience of weed populations to herbicides.

Given the widespread chemical reactivity of 1,4-naphthoquinones *in vivo*, resistant plants might employ NTSR-like strategies, also known as metabolism-based resistance mechanisms (Hatzios, 2004). Catabolic detoxification of herbicides via NTSR strategies in plants broadly mirrors elimination of xenobiotics or drugs in animals. The process occurs over multiple phases through the sequential action of enzymes from a handful of major classes (Riechers *et al.*, 2010). Phase I reactions introduce or unmask reactive functional groups, such as hydroxyls, and often rely on cytochrome P450 enzymes. Phase II metabolism results in the addition of a larger polar group such as GSH or glucose to increase solubility of the compound and involves transferases such as glutathione *S*-transferases (GSTs) or UDP-dependent glycosyl transferases (UGTs). If a sufficiently reactive and available functional group(s) is present on the compound, the detoxification process may not require a phase I enzyme. During phase III, the conjugated compound is shuttled and transported, often via ABC transporters, into the vacuole for storage and/or further degradation, or is incorporated into the cell wall (Gaines *et al.*, 2020).

Similarly to other defensive compounds, such as glucosinolates (Halkier, 2016) and benzoxazinoids (Zhou *et al.*, 2018), 1,4-naphthoquinones are often found stored in their glucosylated forms. In their reduced forms (naphthohydroquinones), the quinone ring of 1,4-naphthoquinones becomes aromatized while its carbonyl groups become hydroxyls available for conjugation with sugars. *Juglans* species, including black and English walnut (Müller and Leistner, 1978), and pecan (*Carya illinoensis*) (Hedin *et al.*, 1980; Gueldner *et al.*, 1994) produce hydrojuglone glucoside. Roots from *I. glandulifera* contain 1,2,4-trihydroxynaphthalene-1-*O*-glucoside, which is the glucosylated form of reduced lawsone (Tříska *et al.*, 2013). Reduced and glucosylated forms of plumbagin and 7-methyljuglone were reported from the common sundew species *Drosera rotundifolia* (Budzianowski, 1996). A Thai medicinal plant, *Diospyros mollis*, produces Makluoside B, which is a glycosylated 1,4-naphthoquinone dimer (Suwama *et al.*, 2018). Thus, just as 1,4-naphthoquinone production evolved independently multiple times, so too must have the glucosylation of reduced forms. This phase II-like process requires the action of oxidoreductases to reduce oxidized quinones and UGTs that remain to be identified. Plants that do not synthesize 1,4-naphthoquinones but that are resistant to their allelopathic effects could theoretically use the same approach. Detection of β-glucosidase activity with high specificity in English walnut husks that catalyzes the release of juglone from its glucosylated form (Duroux *et al.*, 1998) also suggests that 1,4-naphthoquinone-producing plants evolved deglycosylation mechanisms for deploying their stored allelochemicals.

**Fig. 3.**
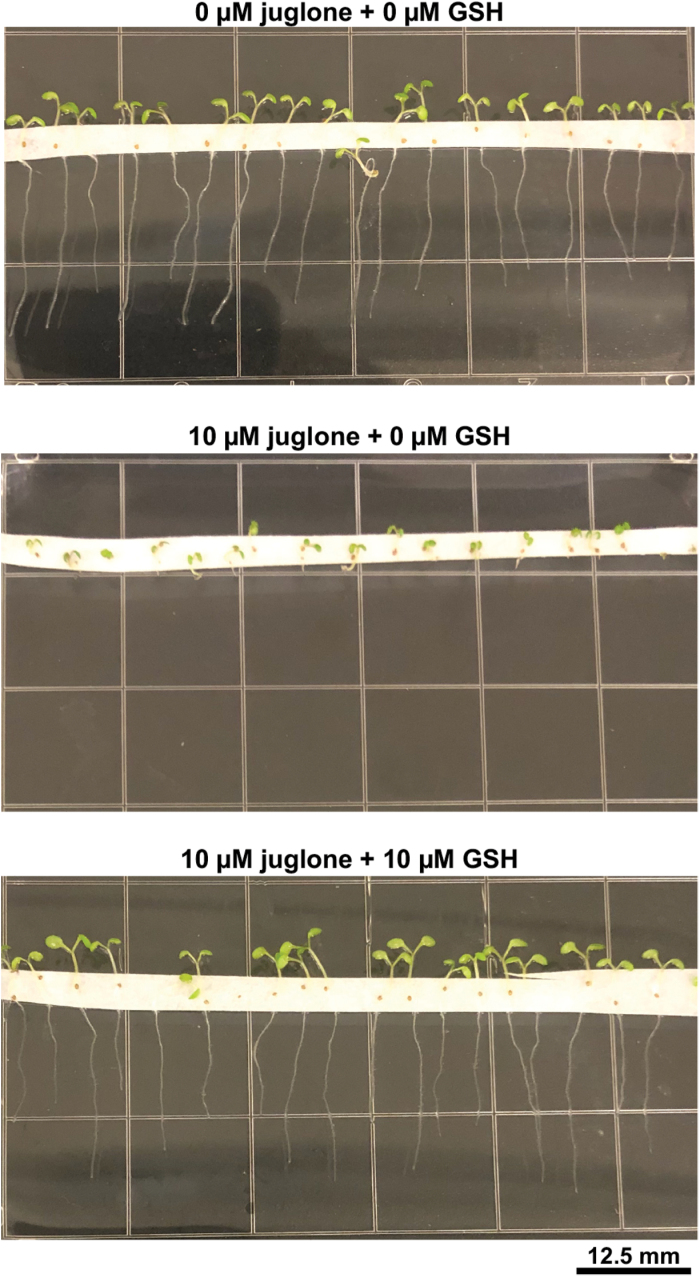
Reduced glutathione (GSH) diminishes the growth inhibitory effects of juglone. Three-day-old wild-type *Arabidopsis thaliana* (Columbia-0) seedlings germinated on 1/2 Murashige and Skoog (MS) medium were transferred to 1/2 MS plates containing 0 µM juglone (top), 10 µM juglone (middle), or 10 µM juglone+10 µM GSH (bottom) for 3 d. Given that GSH spontaneously reacts with juglone to form adducts (Kot *et al.*, 2010), these data indicate that juglone–GSH conjugates are not phytotoxic and/or that they are not taken up by Arabidopsis roots. Therefore, it can be hypothesized that similar to evolved metabolism-based detoxification of certain herbicides (Gaines *et al.*, 2020), conjugation with GSH is one mechanism plants can employ to tolerate the effects of allelopathic 1,4-naphthoquinones such as juglone. Grid squares are 12.5 mm^2^.

Given the propensity of some 1,4-naphthoquinones to react with free thiols (Kot *et al.*, 2010), it can be hypothesized that conjugation with GSH and/or free cysteine might also be a strategy plants use to cope with 1,4-naphthoquinone allelochemicals. Free GSH supplied with juglone *in vitro* is sufficient to reduce the growth inhibitory effects of juglone on *A. thaliana* seedlings (Fig. 3). Thus, even in the absence of a GST, elevated production of GSH *in vivo* could counteract juglone or other structurally similar 1,4-naphthoquinones via direct conjugation for subsequent storage, detoxification, or exudation (Schröder *et al.*, 2007) and/or for neutralizing the effects of any imposed redox stress. Insects (Piskorski and Dorn, 2011; Piskorski *et al.*, 2011), bacteria (Schmidt, 1988), and other organisms have also found ways to detoxify plant 1,4-naphthoquinones so they too may hold clues to mechanisms that resistant plants could employ to manage exposure to the compounds. Bacteria (Lowe *et al.*, 2015) and fungi (Morrissey and Osbourn, 1999) also encounter and detoxify other types of plant allelochemicals, and thus may offer additional lines of investigation.

## Concluding remarks and future directions

Integration of systems biology and functional studies in the context of robust phylogenetic frameworks has shed light on metabolic evolution and plant diversification (Smith *et al.*, 2019). This is perhaps nowhere more apparent than in the recent large-scale biology studies that have revealed insight into the metabolic innovation underlying the evolution of plant 1,4-naphthoquinone pathways. This extraordinary class of compounds arose through both convergent evolution via different pathways and through repeated evolution from the phylloquinone pathway intermediate DHNA. Plants have also evolved a diverse range of mechanisms to deploy 1,4-naphthoquinones into the soil. While it is still not well understood how target plants respond to 1,4-naphthoquinones in the rhizosphere, many plants producing 1,4-naphthoquinones appear to have converged on a strategy to keep the compounds in reduced and glucosylated forms for storage and protection from their allelopathic effects.

Given the propensity of 1,4-naphthoquinones to function as novel chemical weapons in an oxygenic environment and the number of times that they were selected for in nature, there is seemingly great potential for 1,4-naphthoquione-based applications in agriculture. Juglone’s toxicity and impairment of plasma membrane H^+^-ATPase (Rudnicka *et al.*, 2014), for example, gives it a novel herbicidal mode of action unlike any existing synthetic commercial herbicide (Dayan and Duke, 2014). Durán *et al.* (2018) showed that 5-*O*-acyl derivatives of juglone exhibit even greater activity and lipophilicity, further illustrating juglone’s potential as a lead for developing natural product-inspired herbicides. The ability for juglone to react with free thiol groups also makes it a good scaffold for developing natural product-based inhibitors to manage extracellular urease activity in soils (Kot *et al.*, 2010). Harnessing juglone or any other plant 1,4-naphthoquinones as agrochemicals, and to have them produced in crops using synthetic biology, will require continued investment in creating knowledge about biosynthesis, trafficking, and resistance mechanisms. While hairy root culturing is used to study some 1,4-naphthoquinone-producing plants (Gangopadhyay *et al.*, 2011; Yamazaki *et al.*, 2013; Auber *et al.*, 2020), genetic systems for investigating 1,4-naphthoquinone metabolism remain to be developed in many species. Transient infiltration in *Nicotiana tabacum* (Henry *et al.*, 2018) and *N. benthamiana* (Nett *et al.*, 2020) leaves has also emerged as a strong tool in pathway discovery for synthetic biology. Engineering strategies relying on metabolic connections with respiratory or photosynthetic quinones to supply biosynthetic precursors will also require additional study into the factors regulating flux distribution between primary and specialized quinone metabolism. With the recent discovery of a leucine-rich repeat receptor-like kinase (LRR-RLK) involved in quinone-mediated signaling in non-parasitic plant immunity (Laohavisit *et al.*, 2020), there are now also new avenues of inquiry to pursue surrounding the roles of plant 1,4-naphthoquinones in rhizosphere signaling.
